# Supplementary material for: Modeling glioblastoma heterogeneity as a dynamic network of cell states
Source: Mol Syst Biol. 2021 Sep 16;17(9):e10105. doi: 10.15252/msb.202010105 (PMC8444284; doi:10.15252/msb.202010105)
Supplement: Supplementary file 6 — Source Data for Figure 5 [file MSB-17-e10105-s004.zip › Figure5A_sourcedata/GSEA_3071/nosigdb_stateI.GseaPreranked.1622795145664/RICHARDS_NOWAKOWSKI_SCIENCE_2017_MURAL_UPREG.html]

Details for gene set RICHARDS\_NOWAKOWSKI\_SCIENCE\_2017\_MURAL\_UPREG[GSEA]

|  || Dataset | state13071 |
| Phenotype | NoPhenotypeAvailable |
| Upregulated in class | na\_pos |
| GeneSet | RICHARDS\_NOWAKOWSKI\_SCIENCE\_2017\_MURAL\_UPREG |
| Enrichment Score (ES) | 0.4503708 |
| Normalized Enrichment Score (NES) | 3.27872 |
| Nominal p-value | 0.0 |
| FDR q-value | 0.0 |
| FWER p-Value | 0.0 |
Table: GSEA Results Summary

  

Fig 1: Enrichment plot: RICHARDS\_NOWAKOWSKI\_SCIENCE\_2017\_MURAL\_UPREG      
 Profile of the Running ES Score & Positions of GeneSet Members on the Rank Ordered List

  

| PROBE | GENE SYMBOL | GENE\_TITLE | RANK IN GENE LIST | RANK METRIC SCORE | RUNNING ES | CORE ENRICHMENT || 1 | MGP |  |  | 1 | 2.652 | 0.0117 | Yes |
| 2 | COL3A1 |  |  | 3 | 2.646 | 0.0234 | Yes |
| 3 | LUM |  |  | 4 | 2.203 | 0.0334 | Yes |
| 4 | DCN |  |  | 5 | 2.010 | 0.0426 | Yes |
| 5 | PDGFRB |  |  | 9 | 1.893 | 0.0502 | Yes |
| 6 | COL6A1 |  |  | 10 | 1.797 | 0.0584 | Yes |
| 7 | FN1 |  |  | 11 | 1.769 | 0.0664 | Yes |
| 8 | COL5A1 |  |  | 14 | 1.706 | 0.0735 | Yes |
| 9 | PCOLCE |  |  | 15 | 1.696 | 0.0813 | Yes |
| 10 | NUPR1 |  |  | 21 | 1.585 | 0.0869 | Yes |
| 11 | BGN |  |  | 24 | 1.568 | 0.0934 | Yes |
| 12 | MMP14 |  |  | 27 | 1.526 | 0.0997 | Yes |
| 13 | COL4A1 |  |  | 28 | 1.524 | 0.1066 | Yes |
| 14 | CFH |  |  | 29 | 1.523 | 0.1135 | Yes |
| 15 | ITIH5 |  |  | 35 | 1.473 | 0.1186 | Yes |
| 16 | GPRC5C |  |  | 42 | 1.413 | 0.1231 | Yes |
| 17 | IGFBP7 |  |  | 45 | 1.387 | 0.1288 | Yes |
| 18 | TIMP1 |  |  | 47 | 1.381 | 0.1348 | Yes |
| 19 | COL5A2 |  |  | 48 | 1.373 | 0.1410 | Yes |
| 20 | SLC29A1 |  |  | 50 | 1.357 | 0.1469 | Yes |
| 21 | SLC9A3R1 |  |  | 57 | 1.322 | 0.1510 | Yes |
| 22 | CDH11 |  |  | 59 | 1.296 | 0.1565 | Yes |
| 23 | EDIL3 |  |  | 62 | 1.251 | 0.1616 | Yes |
| 24 | COLEC12 |  |  | 63 | 1.241 | 0.1672 | Yes |
| 25 | TFPI |  |  | 64 | 1.241 | 0.1729 | Yes |
| 26 | OLFML2A |  |  | 68 | 1.227 | 0.1775 | Yes |
| 27 | ITGA10 |  |  | 70 | 1.217 | 0.1827 | Yes |
| 28 | COL4A2 |  |  | 73 | 1.203 | 0.1875 | Yes |
| 29 | CD248 |  |  | 79 | 1.190 | 0.1914 | Yes |
| 30 | ID3 |  |  | 86 | 1.152 | 0.1947 | Yes |
| 31 | ASPN |  |  | 92 | 1.117 | 0.1982 | Yes |
| 32 | THY1 |  |  | 94 | 1.114 | 0.2029 | Yes |
| 33 | ZFP36L1 |  |  | 97 | 1.096 | 0.2073 | Yes |
| 34 | NOTCH3 |  |  | 98 | 1.096 | 0.2123 | Yes |
| 35 | DDR2 |  |  | 104 | 1.075 | 0.2156 | Yes |
| 36 | F2R |  |  | 105 | 1.067 | 0.2204 | Yes |
| 37 | SPARC |  |  | 106 | 1.067 | 0.2252 | Yes |
| 38 | SLC2A3 |  |  | 109 | 1.060 | 0.2294 | Yes |
| 39 | S1PR3 |  |  | 114 | 1.043 | 0.2329 | Yes |
| 40 | RASL12 |  |  | 115 | 1.037 | 0.2376 | Yes |
| 41 | SVIL |  |  | 120 | 1.014 | 0.2410 | Yes |
| 42 | NEAT1 |  |  | 123 | 0.982 | 0.2448 | Yes |
| 43 | OLFML3 |  |  | 125 | 0.971 | 0.2489 | Yes |
| 44 | CTSB |  |  | 129 | 0.964 | 0.2523 | Yes |
| 45 | ITPRIPL2 |  |  | 130 | 0.962 | 0.2567 | Yes |
| 46 | UNC5B |  |  | 134 | 0.956 | 0.2601 | Yes |
| 47 | FBN1 |  |  | 138 | 0.944 | 0.2634 | Yes |
| 48 | ARHGAP26 |  |  | 141 | 0.940 | 0.2671 | Yes |
| 49 | SNAI2 |  |  | 142 | 0.940 | 0.2713 | Yes |
| 50 | OSTC |  |  | 151 | 0.915 | 0.2729 | Yes |
| 51 | SERPINH1 |  |  | 157 | 0.902 | 0.2755 | Yes |
| 52 | ITGA1 |  |  | 160 | 0.891 | 0.2789 | Yes |
| 53 | VCL |  |  | 162 | 0.891 | 0.2826 | Yes |
| 54 | PCDH18 |  |  | 171 | 0.870 | 0.2840 | Yes |
| 55 | FAM114A1 |  |  | 174 | 0.864 | 0.2873 | Yes |
| 56 | LAMA4 |  |  | 176 | 0.863 | 0.2909 | Yes |
| 57 | TNFAIP8 |  |  | 179 | 0.858 | 0.2942 | Yes |
| 58 | TLCD2 |  |  | 188 | 0.847 | 0.2955 | Yes |
| 59 | ECE1 |  |  | 195 | 0.839 | 0.2974 | Yes |
| 60 | SLC38A2 |  |  | 200 | 0.826 | 0.2999 | Yes |
| 61 | CALD1 |  |  | 201 | 0.824 | 0.3036 | Yes |
| 62 | RRBP1 |  |  | 208 | 0.810 | 0.3054 | Yes |
| 63 | MEF2C |  |  | 210 | 0.804 | 0.3088 | Yes |
| 64 | SYNE1 |  |  | 214 | 0.800 | 0.3115 | Yes |
| 65 | EBF1 |  |  | 221 | 0.783 | 0.3131 | Yes |
| 66 | CEBPB |  |  | 222 | 0.782 | 0.3167 | Yes |
| 67 | AEBP1 |  |  | 236 | 0.761 | 0.3160 | Yes |
| 68 | B2M |  |  | 241 | 0.744 | 0.3181 | Yes |
| 69 | MSRB3 |  |  | 242 | 0.743 | 0.3215 | Yes |
| 70 | SYTL2 |  |  | 246 | 0.739 | 0.3239 | Yes |
| 71 | LMNA |  |  | 247 | 0.739 | 0.3273 | Yes |
| 72 | PLAC9 |  |  | 254 | 0.731 | 0.3287 | Yes |
| 73 | COL5A3 |  |  | 258 | 0.727 | 0.3310 | Yes |
| 74 | PRKG1 |  |  | 261 | 0.726 | 0.3337 | Yes |
| 75 | PRSS23 |  |  | 264 | 0.721 | 0.3363 | Yes |
| 76 | GSN |  |  | 265 | 0.720 | 0.3396 | Yes |
| 77 | TWIST1 |  |  | 266 | 0.719 | 0.3429 | Yes |
| 78 | IFITM3 |  |  | 268 | 0.714 | 0.3458 | Yes |
| 79 | PLOD1 |  |  | 271 | 0.713 | 0.3484 | Yes |
| 80 | S100A11 |  |  | 275 | 0.707 | 0.3507 | Yes |
| 81 | RBMS1 |  |  | 285 | 0.697 | 0.3510 | Yes |
| 82 | LGALS3BP |  |  | 286 | 0.696 | 0.3542 | Yes |
| 83 | TAPBP |  |  | 287 | 0.692 | 0.3573 | Yes |
| 84 | P4HA1 |  |  | 291 | 0.688 | 0.3595 | Yes |
| 85 | UACA |  |  | 296 | 0.679 | 0.3613 | Yes |
| 86 | CAST |  |  | 298 | 0.675 | 0.3640 | Yes |
| 87 | KANK2 |  |  | 299 | 0.673 | 0.3671 | Yes |
| 88 | MAGED2 |  |  | 302 | 0.669 | 0.3695 | Yes |
| 89 | MYOF |  |  | 310 | 0.663 | 0.3703 | Yes |
| 90 | TPM2 |  |  | 320 | 0.655 | 0.3704 | Yes |
| 91 | SLC3A2 |  |  | 324 | 0.651 | 0.3724 | Yes |
| 92 | VSTM4 |  |  | 326 | 0.649 | 0.3751 | Yes |
| 93 | ISG15 |  |  | 328 | 0.647 | 0.3777 | Yes |
| 94 | COL18A1 |  |  | 336 | 0.638 | 0.3784 | Yes |
| 95 | HSPA5 |  |  | 337 | 0.638 | 0.3813 | Yes |
| 96 | BMP1 |  |  | 346 | 0.631 | 0.3816 | Yes |
| 97 | DYRK2 |  |  | 353 | 0.628 | 0.3826 | Yes |
| 98 | WLS |  |  | 360 | 0.625 | 0.3835 | Yes |
| 99 | ITGB1 |  |  | 371 | 0.615 | 0.3831 | Yes |
| 100 | PLTP |  |  | 374 | 0.611 | 0.3853 | Yes |
| 101 | TRIM38 |  |  | 386 | 0.602 | 0.3845 | Yes |
| 102 | ANXA5 |  |  | 392 | 0.597 | 0.3856 | Yes |
| 103 | CXXC5 |  |  | 393 | 0.594 | 0.3883 | Yes |
| 104 | PDZD2 |  |  | 397 | 0.589 | 0.3901 | Yes |
| 105 | TNS3 |  |  | 399 | 0.588 | 0.3924 | Yes |
| 106 | MGLL |  |  | 406 | 0.581 | 0.3932 | Yes |
| 107 | LAMB1 |  |  | 415 | 0.577 | 0.3933 | Yes |
| 108 | MYL9 |  |  | 417 | 0.576 | 0.3956 | Yes |
| 109 | ARHGAP24 |  |  | 425 | 0.573 | 0.3959 | Yes |
| 110 | NTM |  |  | 427 | 0.572 | 0.3982 | Yes |
| 111 | KLF2 |  |  | 428 | 0.571 | 0.4008 | Yes |
| 112 | CTSL |  |  | 440 | 0.558 | 0.3999 | Yes |
| 113 | MYO1B |  |  | 441 | 0.556 | 0.4024 | Yes |
| 114 | PPIB |  |  | 442 | 0.555 | 0.4049 | Yes |
| 115 | SEL1L |  |  | 448 | 0.549 | 0.4058 | Yes |
| 116 | JUNB |  |  | 454 | 0.546 | 0.4067 | Yes |
| 117 | SSR4 |  |  | 459 | 0.543 | 0.4079 | Yes |
| 118 | SP100 |  |  | 466 | 0.538 | 0.4085 | Yes |
| 119 | KTN1 |  |  | 476 | 0.530 | 0.4080 | Yes |
| 120 | EDNRA |  |  | 479 | 0.529 | 0.4098 | Yes |
| 121 | DAD1 |  |  | 486 | 0.524 | 0.4103 | Yes |
| 122 | LIMS1 |  |  | 498 | 0.514 | 0.4091 | Yes |
| 123 | TMEM263 |  |  | 502 | 0.509 | 0.4105 | Yes |
| 124 | DNAJB4 |  |  | 503 | 0.507 | 0.4128 | Yes |
| 125 | FSTL1 |  |  | 505 | 0.504 | 0.4148 | Yes |
| 126 | LAMA2 |  |  | 506 | 0.503 | 0.4171 | Yes |
| 127 | ARHGAP29 |  |  | 508 | 0.502 | 0.4190 | Yes |
| 128 | TSC22D3 |  |  | 513 | 0.500 | 0.4200 | Yes |
| 129 | DNAJC3 |  |  | 533 | 0.489 | 0.4162 | Yes |
| 130 | PDLIM1 |  |  | 539 | 0.487 | 0.4169 | Yes |
| 131 | HNMT |  |  | 540 | 0.487 | 0.4191 | Yes |
| 132 | VMP1 |  |  | 545 | 0.483 | 0.4200 | Yes |
| 133 | LAPTM4A |  |  | 553 | 0.478 | 0.4200 | Yes |
| 134 | TJP1 |  |  | 557 | 0.477 | 0.4212 | Yes |
| 135 | HSPA1A |  |  | 566 | 0.473 | 0.4208 | Yes |
| 136 | TMEM50A |  |  | 577 | 0.466 | 0.4197 | Yes |
| 137 | FKBP9 |  |  | 590 | 0.458 | 0.4180 | Yes |
| 138 | ENDOD1 |  |  | 592 | 0.457 | 0.4198 | Yes |
| 139 | SERINC3 |  |  | 593 | 0.456 | 0.4219 | Yes |
| 140 | UTRN |  |  | 604 | 0.449 | 0.4207 | Yes |
| 141 | PDE5A |  |  | 608 | 0.447 | 0.4218 | Yes |
| 142 | RHOB |  |  | 609 | 0.447 | 0.4238 | Yes |
| 143 | ETS2 |  |  | 611 | 0.446 | 0.4256 | Yes |
| 144 | PLS3 |  |  | 612 | 0.446 | 0.4276 | Yes |
| 145 | CD63 |  |  | 618 | 0.444 | 0.4280 | Yes |
| 146 | TMOD3 |  |  | 626 | 0.442 | 0.4278 | Yes |
| 147 | LAMC1 |  |  | 640 | 0.439 | 0.4257 | Yes |
| 148 | KLF6 |  |  | 647 | 0.433 | 0.4257 | Yes |
| 149 | EGR1 |  |  | 652 | 0.432 | 0.4264 | Yes |
| 150 | YAP1 |  |  | 657 | 0.430 | 0.4271 | Yes |
| 151 | CD99 |  |  | 659 | 0.430 | 0.4288 | Yes |
| 152 | RSU1 |  |  | 660 | 0.430 | 0.4307 | Yes |
| 153 | APOL2 |  |  | 666 | 0.424 | 0.4311 | Yes |
| 154 | PSMB8 |  |  | 669 | 0.423 | 0.4323 | Yes |
| 155 | AGTRAP |  |  | 676 | 0.422 | 0.4324 | Yes |
| 156 | CPE |  |  | 680 | 0.420 | 0.4333 | Yes |
| 157 | FMNL3 |  |  | 685 | 0.419 | 0.4340 | Yes |
| 158 | PXDN |  |  | 690 | 0.416 | 0.4346 | Yes |
| 159 | EPS8 |  |  | 691 | 0.414 | 0.4365 | Yes |
| 160 | MYADM |  |  | 697 | 0.413 | 0.4368 | Yes |
| 161 | MYH9 |  |  | 701 | 0.411 | 0.4377 | Yes |
| 162 | FERMT2 |  |  | 704 | 0.410 | 0.4389 | Yes |
| 163 | SPATS2L |  |  | 710 | 0.408 | 0.4392 | Yes |
| 164 | BHLHE40 |  |  | 720 | 0.405 | 0.4382 | Yes |
| 165 | TBXA2R |  |  | 722 | 0.404 | 0.4397 | Yes |
| 166 | FKBP10 |  |  | 723 | 0.404 | 0.4415 | Yes |
| 167 | TLN1 |  |  | 731 | 0.402 | 0.4411 | Yes |
| 168 | ARHGAP42 |  |  | 733 | 0.402 | 0.4426 | Yes |
| 169 | PIP4K2A |  |  | 736 | 0.401 | 0.4438 | Yes |
| 170 | KDELR2 |  |  | 737 | 0.401 | 0.4456 | Yes |
| 171 | NR2F2 |  |  | 741 | 0.400 | 0.4465 | Yes |
| 172 | KLF4 |  |  | 742 | 0.400 | 0.4483 | Yes |
| 173 | ARPC1B |  |  | 743 | 0.400 | 0.4501 | Yes |
| 174 | DDIT3 |  |  | 749 | 0.398 | 0.4504 | Yes |
| 175 | IFIT3 |  |  | 767 | 0.393 | 0.4468 | No |
| 176 | PON2 |  |  | 770 | 0.392 | 0.4479 | No |
| 177 | ADAM12 |  |  | 776 | 0.390 | 0.4481 | No |
| 178 | ATP6V0E1 |  |  | 788 | 0.386 | 0.4464 | No |
| 179 | JAG1 |  |  | 796 | 0.383 | 0.4459 | No |
| 180 | HSP90B1 |  |  | 798 | 0.382 | 0.4473 | No |
| 181 | CSPG4 |  |  | 802 | 0.381 | 0.4481 | No |
| 182 | ARL1 |  |  | 813 | 0.377 | 0.4467 | No |
| 183 | ARHGEF17 |  |  | 822 | 0.374 | 0.4458 | No |
| 184 | ALX4 |  |  | 823 | 0.374 | 0.4475 | No |
| 185 | FOS |  |  | 832 | 0.370 | 0.4467 | No |
| 186 | HBP1 |  |  | 833 | 0.370 | 0.4484 | No |
| 187 | IFITM2 |  |  | 836 | 0.370 | 0.4494 | No |
| 188 | SLC40A1 |  |  | 848 | 0.366 | 0.4476 | No |
| 189 | FOSB |  |  | 854 | 0.364 | 0.4476 | No |
| 190 | RNF152 |  |  | 861 | 0.362 | 0.4474 | No |
| 191 | MRC2 |  |  | 863 | 0.361 | 0.4487 | No |
| 192 | HTRA3 |  |  | 884 | 0.354 | 0.4440 | No |
| 193 | ITGA11 |  |  | 885 | 0.353 | 0.4456 | No |
| 194 | SGCE |  |  | 891 | 0.353 | 0.4456 | No |
| 195 | MIR22HG |  |  | 894 | 0.352 | 0.4466 | No |
| 196 | PAPSS2 |  |  | 898 | 0.351 | 0.4472 | No |
| 197 | SLC12A2 |  |  | 903 | 0.350 | 0.4476 | No |
| 198 | COMT |  |  | 906 | 0.350 | 0.4485 | No |
| 199 | PARP8 |  |  | 907 | 0.350 | 0.4501 | No |
| 200 | IFI6 |  |  | 923 | 0.344 | 0.4469 | No |
| 201 | EVI5 |  |  | 933 | 0.343 | 0.4456 | No |
| 202 | DYNLT3 |  |  | 946 | 0.340 | 0.4434 | No |
| 203 | PXDC1 |  |  | 952 | 0.339 | 0.4433 | No |
| 204 | SP140L |  |  | 956 | 0.338 | 0.4439 | No |
| 205 | DOCK10 |  |  | 957 | 0.338 | 0.4454 | No |
| 206 | TPT1 |  |  | 971 | 0.334 | 0.4428 | No |
| 207 | OXR1 |  |  | 979 | 0.332 | 0.4421 | No |
| 208 | ZFHX3 |  |  | 1001 | 0.327 | 0.4370 | No |
| 209 | PARP14 |  |  | 1026 | 0.323 | 0.4308 | No |
| 210 | RNF213 |  |  | 1027 | 0.322 | 0.4323 | No |
| 211 | LGMN |  |  | 1031 | 0.321 | 0.4328 | No |
| 212 | CALCOCO2 |  |  | 1038 | 0.320 | 0.4324 | No |
| 213 | NBPF14 |  |  | 1045 | 0.319 | 0.4319 | No |
| 214 | MYL12A |  |  | 1053 | 0.316 | 0.4311 | No |
| 215 | CD81 |  |  | 1059 | 0.315 | 0.4310 | No |
| 216 | IFI16 |  |  | 1062 | 0.315 | 0.4318 | No |
| 217 | SERINC5 |  |  | 1066 | 0.313 | 0.4323 | No |
| 218 | PMP22 |  |  | 1069 | 0.312 | 0.4330 | No |
| 219 | TBX15 |  |  | 1074 | 0.312 | 0.4332 | No |
| 220 | EPAS1 |  |  | 1078 | 0.311 | 0.4337 | No |
| 221 | KDELR1 |  |  | 1090 | 0.309 | 0.4316 | No |
| 222 | KIAA1109 |  |  | 1093 | 0.308 | 0.4323 | No |
| 223 | S1PR2 |  |  | 1104 | 0.305 | 0.4306 | No |
| 224 | GSTK1 |  |  | 1113 | 0.304 | 0.4294 | No |
| 225 | PHACTR2 |  |  | 1142 | 0.299 | 0.4219 | No |
| 226 | JAK1 |  |  | 1145 | 0.299 | 0.4226 | No |
| 227 | CYTH3 |  |  | 1157 | 0.297 | 0.4205 | No |
| 228 | NR3C1 |  |  | 1162 | 0.297 | 0.4206 | No |
| 229 | NUMB |  |  | 1167 | 0.295 | 0.4207 | No |
| 230 | EPB41L2 |  |  | 1170 | 0.295 | 0.4214 | No |
| 231 | NEK7 |  |  | 1176 | 0.293 | 0.4211 | No |
| 232 | ESYT2 |  |  | 1183 | 0.292 | 0.4205 | No |
| 233 | MYLK |  |  | 1204 | 0.287 | 0.4155 | No |
| 234 | IL6ST |  |  | 1215 | 0.285 | 0.4136 | No |
| 235 | REST |  |  | 1225 | 0.285 | 0.4121 | No |
| 236 | ARSG |  |  | 1239 | 0.280 | 0.4092 | No |
| 237 | AFAP1L2 |  |  | 1247 | 0.278 | 0.4083 | No |
| 238 | ADD3 |  |  | 1261 | 0.275 | 0.4054 | No |
| 239 | PHLDA1 |  |  | 1262 | 0.275 | 0.4067 | No |
| 240 | ATL3 |  |  | 1270 | 0.274 | 0.4057 | No |
| 241 | HADHB |  |  | 1271 | 0.274 | 0.4069 | No |
| 242 | LEPROT |  |  | 1278 | 0.272 | 0.4063 | No |
| 243 | ATP8B1 |  |  | 1280 | 0.272 | 0.4072 | No |
| 244 | TNKS1BP1 |  |  | 1294 | 0.269 | 0.4043 | No |
| 245 | SDHD |  |  | 1299 | 0.269 | 0.4042 | No |
| 246 | PARVA |  |  | 1301 | 0.268 | 0.4051 | No |
| 247 | PLOD2 |  |  | 1304 | 0.268 | 0.4057 | No |
| 248 | CPED1 |  |  | 1309 | 0.267 | 0.4057 | No |
| 249 | CEBPD |  |  | 1310 | 0.267 | 0.4069 | No |
| 250 | FOXP1 |  |  | 1319 | 0.265 | 0.4056 | No |
| 251 | CAV1 |  |  | 1341 | 0.261 | 0.4001 | No |
| 252 | ZFP36L2 |  |  | 1378 | 0.255 | 0.3899 | No |
| 253 | MSN |  |  | 1379 | 0.255 | 0.3910 | No |
| 254 | WDR41 |  |  | 1407 | 0.251 | 0.3836 | No |
| 255 | TGFBR2 |  |  | 1410 | 0.251 | 0.3841 | No |
| 256 | CYSTM1 |  |  | 1411 | 0.250 | 0.3853 | No |
| 257 | CHP1 |  |  | 1429 | -0.251 | 0.3810 | No |
| 258 | CD44 |  |  | 1430 | -0.251 | 0.3822 | No |
| 259 | GAPDH |  |  | 1459 | -0.253 | 0.3744 | No |
| 260 | UQCRQ |  |  | 1464 | -0.254 | 0.3743 | No |
| 261 | FARP1 |  |  | 1496 | -0.257 | 0.3657 | No |
| 262 | LIN7A |  |  | 1573 | -0.264 | 0.3428 | No |
| 263 | CNP |  |  | 1582 | -0.265 | 0.3415 | No |
| 264 | RPL41 |  |  | 1600 | -0.267 | 0.3373 | No |
| 265 | KCNJ8 |  |  | 1601 | -0.268 | 0.3385 | No |
| 266 | ASNA1 |  |  | 1629 | -0.270 | 0.3312 | No |
| 267 | STARD13 |  |  | 1672 | -0.276 | 0.3192 | No |
| 268 | COX6B1 |  |  | 1673 | -0.277 | 0.3204 | No |
| 269 | PRICKLE2 |  |  | 1683 | -0.277 | 0.3188 | No |
| 270 | ISYNA1 |  |  | 1692 | -0.278 | 0.3176 | No |
| 271 | DENND2A |  |  | 1699 | -0.279 | 0.3169 | No |
| 272 | POLR2L |  |  | 1742 | -0.285 | 0.3049 | No |
| 273 | EEF1D |  |  | 1769 | -0.290 | 0.2980 | No |
| 274 | APOLD1 |  |  | 1781 | -0.291 | 0.2959 | No |
| 275 | CDK14 |  |  | 1798 | -0.293 | 0.2921 | No |
| 276 | A2M |  |  | 1808 | -0.295 | 0.2906 | No |
| 277 | TPI1 |  |  | 1828 | -0.297 | 0.2859 | No |
| 278 | RHOA |  |  | 1843 | -0.298 | 0.2829 | No |
| 279 | AGFG1 |  |  | 1858 | -0.300 | 0.2798 | No |
| 280 | FCGRT |  |  | 1871 | -0.301 | 0.2774 | No |
| 281 | HEY2 |  |  | 1882 | -0.302 | 0.2756 | No |
| 282 | SPCS1 |  |  | 1887 | -0.303 | 0.2757 | No |
| 283 | PLCE1 |  |  | 1890 | -0.303 | 0.2764 | No |
| 284 | TACC1 |  |  | 1916 | -0.307 | 0.2699 | No |
| 285 | MRPL33 |  |  | 1978 | -0.315 | 0.2520 | No |
| 286 | FOSL2 |  |  | 2015 | -0.319 | 0.2421 | No |
| 287 | MRPL36 |  |  | 2023 | -0.321 | 0.2413 | No |
| 288 | STT3B |  |  | 2029 | -0.322 | 0.2412 | No |
| 289 | COBLL1 |  |  | 2079 | -0.331 | 0.2272 | No |
| 290 | ETS1 |  |  | 2086 | -0.332 | 0.2268 | No |
| 291 | NDUFA11 |  |  | 2097 | -0.334 | 0.2251 | No |
| 292 | PMEPA1 |  |  | 2107 | -0.336 | 0.2238 | No |
| 293 | C18orf54 |  |  | 2128 | -0.339 | 0.2190 | No |
| 294 | CALCRL |  |  | 2249 | -0.363 | 0.1827 | No |
| 295 | GLO1 |  |  | 2292 | -0.371 | 0.1710 | No |
| 296 | PFN1 |  |  | 2305 | -0.373 | 0.1689 | No |
| 297 | MCAM |  |  | 2310 | -0.374 | 0.1694 | No |
| 298 | BTG2 |  |  | 2318 | -0.375 | 0.1689 | No |
| 299 | SEPT4 |  |  | 2344 | -0.383 | 0.1627 | No |
| 300 | PAG1 |  |  | 2347 | -0.383 | 0.1638 | No |
| 301 | RBM6 |  |  | 2403 | -0.393 | 0.1482 | No |
| 302 | CD9 |  |  | 2444 | -0.400 | 0.1373 | No |
| 303 | PLAT |  |  | 2501 | -0.415 | 0.1215 | No |
| 304 | TLE1 |  |  | 2502 | -0.415 | 0.1234 | No |
| 305 | ST3GAL5 |  |  | 2526 | -0.421 | 0.1180 | No |
| 306 | GNG11 |  |  | 2554 | -0.427 | 0.1114 | No |
| 307 | LDHB |  |  | 2597 | -0.438 | 0.1001 | No |
| 308 | UQCR11 |  |  | 2614 | -0.443 | 0.0970 | No |
| 309 | SRSF3 |  |  | 2631 | -0.447 | 0.0940 | No |
| 310 | JAM3 |  |  | 2654 | -0.455 | 0.0891 | No |
| 311 | JUND |  |  | 2670 | -0.464 | 0.0864 | No |
| 312 | SLC43A3 |  |  | 2713 | -0.475 | 0.0753 | No |
| 313 | SLC25A5 |  |  | 2765 | -0.493 | 0.0614 | No |
| 314 | COX8A |  |  | 2819 | -0.509 | 0.0469 | No |
| 315 | COX6C |  |  | 2825 | -0.511 | 0.0477 | No |
| 316 | PLXDC1 |  |  | 2835 | -0.514 | 0.0471 | No |
| 317 | ADAMTS1 |  |  | 2841 | -0.517 | 0.0479 | No |
| 318 | TANC1 |  |  | 2878 | -0.531 | 0.0389 | No |
| 319 | CACNB2 |  |  | 2883 | -0.531 | 0.0401 | No |
| 320 | AMOTL1 |  |  | 2885 | -0.533 | 0.0422 | No |
| 321 | SPECC1 |  |  | 2909 | -0.546 | 0.0374 | No |
| 322 | MT2A |  |  | 2910 | -0.547 | 0.0399 | No |
| 323 | SFTA1P |  |  | 2915 | -0.550 | 0.0411 | No |
| 324 | SPRY4 |  |  | 2916 | -0.552 | 0.0436 | No |
| 325 | DAG1 |  |  | 2938 | -0.566 | 0.0395 | No |
| 326 | NT5DC2 |  |  | 2941 | -0.568 | 0.0415 | No |
| 327 | ITPR2 |  |  | 3002 | -0.603 | 0.0252 | No |
| 328 | TNFRSF21 |  |  | 3004 | -0.604 | 0.0276 | No |
| 329 | RBPMS |  |  | 3015 | -0.610 | 0.0272 | No |
| 330 | ITPR1 |  |  | 3029 | -0.619 | 0.0259 | No |
| 331 | SERPINE1 |  |  | 3050 | -0.634 | 0.0225 | No |
| 332 | LMO2 |  |  | 3051 | -0.635 | 0.0254 | No |
| 333 | AP2M1 |  |  | 3070 | -0.651 | 0.0226 | No |
| 334 | RBP1 |  |  | 3091 | -0.667 | 0.0193 | No |
| 335 | RGS16 |  |  | 3134 | -0.708 | 0.0092 | No |
| 336 | NDUFAF3 |  |  | 3150 | -0.728 | 0.0078 | No |
| 337 | SPRY1 |  |  | 3163 | -0.746 | 0.0074 | No |
| 338 | CCDC102B |  |  | 3221 | -0.807 | -0.0070 | No |
| 339 | BCHE |  |  | 3236 | -0.825 | -0.0077 | No |
| 340 | INPP4B |  |  | 3263 | -0.871 | -0.0120 | No |
| 341 | SLC1A3 |  |  | 3277 | -0.889 | -0.0120 | No |
| 342 | KLHL23 |  |  | 3287 | -0.907 | -0.0108 | No |
| 343 | PTPRG |  |  | 3308 | -0.945 | -0.0128 | No |
| 344 | EMP2 |  |  | 3338 | -0.998 | -0.0175 | No |
| 345 | NINJ1 |  |  | 3372 | -1.108 | -0.0229 | No |
| 346 | ADAMTS9 |  |  | 3407 | -1.260 | -0.0279 | No |
| 347 | LIMA1 |  |  | 3429 | -1.353 | -0.0284 | No |
| 348 | NBL1 |  |  | 3432 | -1.368 | -0.0228 | No |
| 349 | AKAP12 |  |  | 3444 | -1.501 | -0.0195 | No |
| 350 | MAP3K1 |  |  | 3449 | -1.546 | -0.0137 | No |
| 351 | TSPAN12 |  |  | 3451 | -1.579 | -0.0069 | No |
| 352 | CADM4 |  |  | 3468 | -1.784 | -0.0038 | No |
| 353 | PTN |  |  | 3476 | -1.843 | 0.0023 | No |
| 354 | GPM6B |  |  | 3478 | -1.861 | 0.0104 | No |
Table: GSEA details [plain text format]

  

Fig 2: RICHARDS\_NOWAKOWSKI\_SCIENCE\_2017\_MURAL\_UPREG: Random ES distribution      
 Gene set null distribution of ES for **RICHARDS\_NOWAKOWSKI\_SCIENCE\_2017\_MURAL\_UPREG**
